# D-2-Hydroxyglutarate does not mimic all the IDH mutation effects, in particular the reduced etoposide-triggered apoptosis mediated by an alteration in mitochondrial NADH

**DOI:** 10.1038/cddis.2015.13

**Published:** 2015-03-26

**Authors:** K Oizel, C Gratas, A Nadaradjane, L Oliver, F M Vallette, C Pecqueur

**Affiliations:** 1 CRCNA - INSERM UMR 892 - CNRS UMR 6299, Nantes F44007, France; 2 Faculté de Médecine, Université de Nantes, Nantes F44007, France; 3 Centre Hospitalier-Universitaire (CHU) de Nantes, Nantes F44093, France; 4 Institut de Cancérologie de l'Ouest - René Gauducheau, St Herblain F44805, France

## Abstract

Somatic mutations in isocitrate dehydrogenase (IDH)-1 and -2 have recently been described in glioma. This mutation leads to a neomorphic enzymatic activity as the conversion of isocitrate to alpha ketoglutarate (*α*KG) is replaced by the conversion of *α*KG to D-2-hydroxyglutarate (D-2HG) with NADPH oxidation. It has been suggested that this oncometabolite D-2HG via inhibition of *α*KG-dioxygenases is involved in multiple functions such as epigenetic modifications or hypoxia responses. The present study is aimed at deciphering how the mutant IDH can affect cancer pathogenesis, in particular with respect to its associated oncometabolite D-2HG. We show that the overexpression of mutant IDH in glioma cells or treatment with D-2HG triggered an increase in cell proliferation. However, although mutant IDH reduced cell sensitivity to the apoptotic inducer etoposide, D-2HG exhibited no effect on apoptosis. Instead, we found that the apoptotic effect was mediated through the mitochondrial NADH pool reduction and could be inhibited by oxamate. These data show that besides D-2HG production, mutant IDH affects other crucial metabolite pools. These observations lead to a better understanding of the biology of IDH mutations in gliomas and their response to therapy.

Gliomas are the most common type of human brain tumors and can be classified based on clinical and pathological criteria in four grades. The grade IV glioma, commonly known as glioblastoma multiforme (GBM), is the most invasive form and has a dismal prognosis with <5% patient survival at 5 years. These GBM can develop *de novo* (primary GBM) or through the progression from low-grade tumors (secondary GBM). Although these two types of GBM are histologically similar, primary and secondary GBM exhibit distinct genetic patterns. A recent integrated genome analysis of human GBM shows that 12% of these tumors have a mutation in the gene encoding isocitrate dehydrogenase 1 (IDH1) and to a lesser extent in IDH2 gene.^1^ This mutation is present in >90% secondary GBMs, whereas it is present in <5% primary GBMs.^2^ Mutations in IDH1 and IDH2 have also been identified in acute myeloid leukemia (AML)^3^ and chondrosarcomas.^4^ The occurrence of IDH mutations predicts a significantly longer survival for patients affected by GBM or grade III gliomas.^1, 2^ Whether this difference is driven by IDH mutations or reflects other fundamental biological differences between primary and secondary GBM is, as yet, unclear. For example, the prognostic significance of IDH mutations may be secondary to their prevalence among younger patients, as age is a well-known prognostic factor in gliomas.^5^ In AML, the prognostic significance of IDH mutations is more ambiguous. Several studies have reported that IDH mutations do not affect the prognosis in AML, whereas other studies have found that IDH mutations are associated with an increased or decreased risk of relapse when compared with IDH wild-type patients.^6, 7^

The human genome has five IDH genes coding for three different IDH isoforms, the activities of which depend on either nicotinamide adenine dinucleotide (NAD+) for IDH3 or nicotinamide adenine dinucleotide phosphate (NADP+) for IDH1 and IDH2. Both IDH2 and IDH3 are located in the mitochondria where they participate in the TCA cycle, whereas IDH1 is mostly cytosolic.^8^ To date, all reported mutations are located in the IDH1 and IDH2 genes and result in an amino-acid substitution at residues located in the enzymatic active site, respectively, R132 for IDH1 and R140 or R172 for IDH2. This mutation disrupts the normal enzymatic function of IDH, that is, the conversion of isocitrate to alpha ketoglutarate (*α*KG) with the concomitant production of NADPH. Instead, mutant IDH displays a neomorphic activity converting *α*KG into D-2-hydroxyglutarate (D-2HG), although reducing NADPH.^9^ As a result, mutant IDH may alter the redox state of cells, modulate the activity of metabolic and epigenetic tumor suppressor enzymes that use *α*KG as a co-substrate.^10^ Loss of IDH function may also alter normal mitochondrial function and promote a metabolic switch in cancer cells to glycolysis.^11, 12^

Mutant IDH is widely believed to have the ability to transform cells by modulating *α*KG-dependent enzymes. D-2HG and *α*KG are structurally similar suggesting that D-2HG may act as a competitive inhibitor of *α*KG-dioxygenases including prolyl hydroxylase involved in HIF-1*α* stability, histone demethylases and the Ten-Eleven Translocation (TET) family of 5-methylcytosine hydroxylases involved in epigenetic modifications of DNA.^13, 14^ In fact, IDH mutations lead to numerous metabolic abnormalities besides D-2HG production. Deciphering the relative importance of either D-2HG production, *α*KG or NADPH reduction in cancer pathogenesis remains to be determined. In this paper, we show that mutant IDH increases cell proliferation and reduces etoposide (ETO)-induced cell death through different metabolic pathways. Although cell proliferation changes are mediated through D-2HG, alteration in the mitochondrial NADH pool is involved in the response to apoptosis.

## Results

### Glioma cell lines overexpressing IDH1^R132^ exhibit reduced NADP production and increased cell proliferation

**Figure 1:**
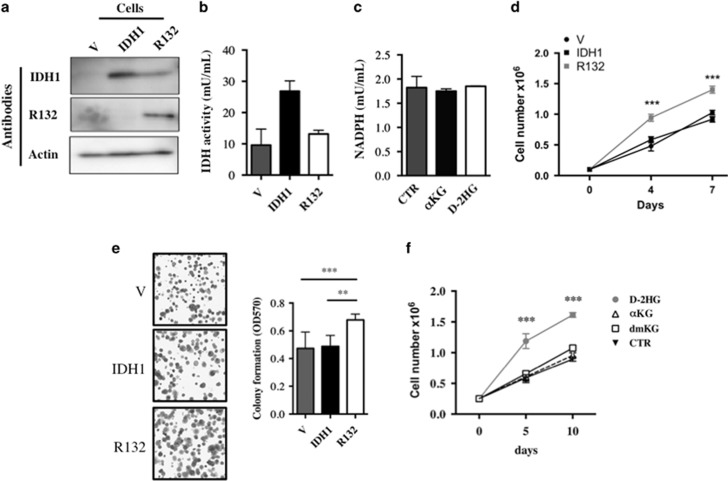
(**a**) Expression of wild-type or mutant IDH1 isoforms in stable overexpressing cells was confirmed by immunoblotting using whole lysates (40 μg). Actin was used as a loading control. (**b**) IDH activity of cells overexpressing wild-type or mutant IDH1 isoforms. Cells (1 × 10^6^) were plated, lysed the next day and subsequently assayed for their IDH activity. (**c**) NADPH production in cells treated for 6 days with *α*KG (3 mM) or D-2HG (3 mM) as in **b**. (**d**) Proliferation of wild-type and mutant IDH1-overexpressing cells. Cells were plated at 1 × 10^5^ cells and counted 3 and 7 days later using trypan blue staining. (**e**) The ability of forming colonies of wild-type and mutant IDH1-overexpressing cells. Cells were plated at 500 cells per well then fixed and stained with violet crystal 1 week later. (**f**) Proliferation of cells treated with *α*KG (3 mM), dimethyl-*α*KG (3 mM) and HG (3 mM). Cells were plated at 1 × 10^5^ cells and counted 5 and 10 days later using trypan blue staining. Results are expressed as the mean±S.E.M. of three experiments performed in triplicate. V, empty vector expressing cells; IDH1, wild-type IDH1-expressing cells; R132, IDH1^R132^-expressing cells transfected. **P<0.01 and ***P<0.001

To date, the lack of mutant IDH glioma cell lines has been an issue in the study of the pathogenic role of IDH mutations. To address this, we stably transfected a human glioma cell line, U251, with vectors encoding the wild-type or the mutant form of IDH1 (IDH1^R132^; Figure 1a). IDH1 was detected by western blot in cytoplasmic lysate. In order to determine whether IDH1^R132^ overexpression was associated with the neomorphic enzymatic activity, the NADPH level was measured in these cells (Figure 1b). As expected, lysate from IDH1-overexpressing cells exhibited higher levels of IDH activity as compared with IDH1^R132^. Note that the level of NADPH detected in control cells was similar to the level observed in mutant IDH-expressing cells. Addition of either *α*KG or D-2HG to the cell medium did not affect the total cellular NADPH level (Figure 1c). In order to study how these mutations can affect cancer pathogenesis, cell proliferation and cell death response to different stimuli were measured. An increased proliferation and an increased ability to form colonies when cells were plated at very low density were detected in cells overexpressing IDH1^R132^ (Figures 1d and e). To determine whether D-2HG had a role in this increased proliferation, cells were cultured for 6 days in the presence of *α*KG, a cell-permeant form of *α*KG, dimethyl *α*KG (dmKG) or D-2HG. As expected, *α*KG and dmKG had no effect on cell proliferation, whereas, similar to the overexpression of IDH1^R132^, D-2HG increased significantly cell proliferation compared with the control or cells treated with the different forms of *α*KG (Figure 1f).

### IDH1^R132^ reduces ETO-induced apoptosis

**Figure 2:**
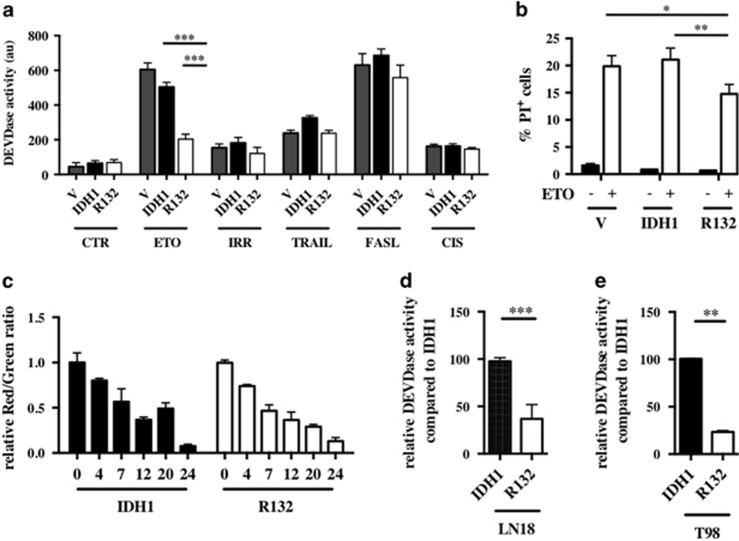
(**a**) Caspase 3 activation was determined with DEVDase activity assay in stable transfected U251 cells expressing empty vector, wild-type and mutant IDH1 isoforms after induction of apoptosis. Cells were plated at 5 × 10^5^ cells and treated the next day with different inducers of cell apoptosis. Cellular extracts were prepared from untreated cells (CTR), 5 h after treatment with TRAIL (50 ng/ml), 24 h after ETO (50 *μ*g/ml), FASL (60ng/ml) or cisplatin (CIS) (15 *μ*g/ml) and 72 h after *γ*-irradiation (IRR; 5 Gy). (**b**) The number of dead cells 24 h after ETO (50 *μ*g/ml) treatment was determined by FACS. Cells were incubated 5 min with propidium iodide (1 *μ*g/ml) and analyzed by FACS. (**c**) The mitochondrial membrane potential was determined by FACS after ETO (50 *μ*g/ml) treatment at different time points. Cells were incubated 15 min with JC-1 probe and analyzed by FACS. (**d** and **e**) Caspase 3 activation after 24 h ETO (50 *μ*g/ml) exposure was determined with DEVDase activity assay, respectively, in wild-type and mutant IDH1-overexpressing LN18 and T98 cells. Results are expressed relative to wild-type IDH1-expressing cells. Results are expressed as the mean±S.E.M. of three experiments performed in triplicate. V, empty vector expressing cells; IDH1, wild-type IDH1-expressing cells; R132, IDH1^R132^-expressing cells transfected. *P<0.05, **P<0.01 and ***P<0.001

Cell death was measured at different time points after irradiation (5 Gy), ETO (50 *μ*g/ml), TRAIL (50 ng/ml), FasL (60 ng/ml) or cisplatin (15 *μ*g/ml) treatment in U251 cells. All treatments were associated with a significant death and activation of caspase-3 (Table 1). However, the optimal time point of cell death induction varied from 6 h with TRAIL to 24 h with ETO, Cisplatin, and FASL treatments, and to 72 h with irradiation. Next, sensitivity to cell death was analyzed in IDH1- and IDH1^R132^-overexpressing cells. For most treatments, overexpression of IDH1^R132^ did not affect caspase-3 activity (Figure 2a). However, although addition of ETO caused a high caspase-3 activation in control and wild-type IDH1-overexpressing cells, activation of caspase-3 was significantly reduced in IDH1^R132^-overexpressing cells. These results were confirmed by FACs analysis, which showed that the percentage of propidium iodide-stained cells after ETO exposure was lower in IDH1^R132^ cells compared with the control and IDH1 cells (Figure 2b). During apoptosis, the integrity of the mitochondrial outer membrane is compromised, a process called mitochondrial outer membrane permeabilization (MOMP). In order to determine whether IDH1 or IDH1^R132^ expression was affecting the MOMP, we measured the mitochondrial membrane potential ΔΨm in our cells using JC-1 staining. ΔΨm was reduced in a time-dependent manner after ETO exposure as indicated by the decrease of the red/green ratio (Figure 2c). Of note, the median of healthy cells red fluorescence was significantly higher in IDH1^R132^ cells compared with IDH1 cells (845±75 *versus* 655±56; *n*=6; *P*<0.05), suggesting a mitochondrial hyperpolarization of IDH1^R132^ cells. Sensitivity to ETO was then tested in other glioma cell lines overexpressing either IDH1 or IDH1^R132^, LN18 and T98 (Figures 2d and e). In both cells lines, IDH1^R132^ overexpression was associated with reduced ETO-induced cell apoptosis.

### Mutant IDH2 decreases sensitivity to ETO but D-2HG has no effect

**Figure 3:**
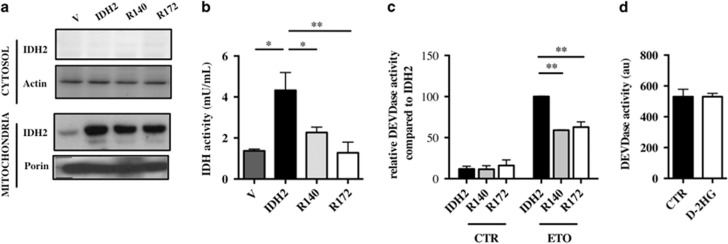
(**a**) Expression of wild-type (IDH2) or mutant IDH2 (R140 and R172) isoforms in stable overexpressing U251 cells was confirmed by immunoblotting using whole lysates (40 μg) or purified mitochondria. Actin and porin were used as a loading control, respectively, for whole lysates and mitochondria. (**b**) IDH activity in cells overexpressing wild-type or mutant IDH2 isoforms. Cells (1 × 10^6^) were plated, lysed the next day and subsequently assayed for their ability to generate NADPH. (**c**) Caspase 3 activation was determined with DEVDase activity assay in stable U251 cells expressing wild-type and mutant IDH2 isoforms after 24 h ETO (50 *μ*g/ml) exposure. Results are expressed relative to wild-type IDH2-expressing cells. (**d**) Caspase 3 activation was determined with DEVDase activity assay after 24 h ETO (50 *μ*g/ml) exposure in U251 cells treated with D-2HG (3 mM) for 6 days. Results are expressed as the mean±S.E.M. of three experiments performed in triplicate. V, empty vector expressing cells; IDH2, wild-type IDH2-expressing cells; R140, IDH1^R140^-expressing cells transfected; R172, IDH1^R172^-expressing cells transfected. *P<0.05, **P<0.01 and ***P<0.001

To determine whether mutant IDH2 triggered similar effects on cell death, U251 cells were transfected with vector, IDH2, IDH2^R140^ and IDH2^R172^. Western blot analysis showed that IDH2 proteins were expressed in the mitochondria and not in the cytosol (Figure 3a). Similarly to IDH1^R132^, mutant IDH2-expressing cells exhibited decreased NADPH consumption corresponding to its neomorphic activity (Figure 3b). Interestingly, caspase-3 activation following ETO treatment was also significantly reduced (Figure 3c). Altogether, these results showed that all mutants IDH, independently of their respective subcellular localization, reduced ETO-induced cell death.

To determine whether D-2HG could be involved in this phenotype, U251 cells were treated with 3 mM or 10 mM of D-2HG for 6 days before ETO treatment. However, none of the pretreatments affected ETO-induced apoptosis (Figure 3d and data not shown). Thus, D-2HG does not fully recapitulate the mutant IDH phenotypes as exogenous D-2HG is able to increase cell proliferation but does not affect cell sensitivity to ETO.

### Reduced mitochondrial spare capacity with IDH1^R132^ but not with D-2HG

**Figure 4:**
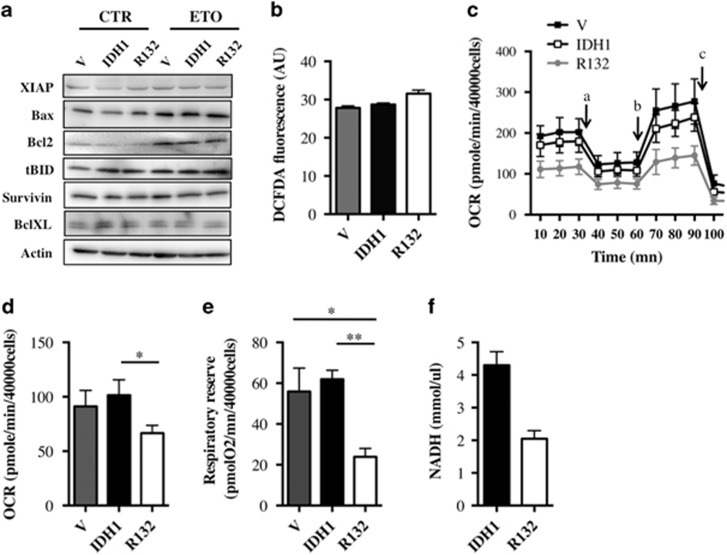
(**a**) Expression of proteins involved in apoptosis in U251 cells expressing empty vector, wild-type and mutant IDH1 isoforms. Whole lysates of cells were isolated 24 h after vehicule (V) or etoposide (ETO) treatment and analyzed (40 *μ*g) by immunoblotting with the indicated antibodies (left panel). Actin was used as a loading control. (**b**) ROS production was measured using the DCFDA probe. U251 cells expressing empty vector, wild-type and mutant IDH1 isoforms were seeded at 2.5 × 10^4^ cells then incubated with the DCFDA probe. Fluorescence was measured at 538 nm every 3 min for 75 min and the slope corresponding to ROS production was calculated. (**c**) Oxygen consumption rate (OCR) of stable cells expressing empty vector, wild-type and mutant IDH1 isoforms was measured over time. Cells (4 × 10^4^) were plated and OCR was measured 24 h later by a XF24 Analyzer (Seahorse Bioscience). Mitochondrial inhibitors (oligomycin(a), CCCP(b), and rotenone and antimycin A(c)) were added as indicated with arrows. (**d**) Basal oxygen consumption was determined by measuring OCR as in **c** removing the non-mitochondrial oxygen consumption (OCR upon rotenone and antimycin A treatment). (**e**) The respiratory reserve of U251 cells expressing empty vector, wild-type and mutant IDH1 isoforms was measured by a XF24 Analyzer (Seahorse Bioscience). The respiratory reserve was determined as the difference between maximal OCR and basal OCR. (**f**) NADH production of cells expressing wild-type or mutant IDH1 isoforms. Cells (1 × 10^6^) were plated, lysed the next day and subsequently assayed for their ability to produce NADH. Results are expressed as the mean±S.E.M. of three experiments performed in triplicate

To better understand how IDH1^R132^ affects apoptosis, several parameters were analyzed. IDH mutation can alter protein expression through epigenetic modifications, redox homeostasis and metabolism of glucose, glutamine (Gln) and fatty acids. As an aberrant methylation status of Bax and Bcl2 has been shown to be associated with apoptosis escape,^15^ expression of these proteins, as well as BclXL, XIAP, truncated BID (tBID) and survivin, was analyzed by western blot (Figure 4a). However, neither IDH1^R132^ nor wild-type IDH1 expression affected protein expression of either control or ETO-treated cells. Expression of Bax and Bcl2 increased upon ETO treatment but to the same extent in vector, IDH1- and IDH1^R132^-expressing cells. Of note, a similar pattern of expression of Bax and Bcl2 was observed in cells treated with *α*KG or D-2HG before ETO (Supplementary Figure S1). As ROS and mitochondria have an important role in apoptosis, we then measured ROS production using the DCFDA probe and cell metabolism using the XF24 analyzer. Cellular ROS were not affected by either wild-type or IDH1^R132^ overexpression as shown in Figure 4b. Of note, ROS production was increased with rotenone, an inhibitor of the mitochondrial complex I able to induce mitochondrial ROS production, and decreased with EGCG, a polyphenol compound known for its antioxidant effect (Supplementary Figure S2). Interestingly, overexpression of IDH1^R132^ and IDH2^R172^ was associated with a slight but significant decreased in mitochondrial oxygen consumption rate (OCR) (Figures 4c and d, Supplementary Figure S3A). In addition to the basal OCR, maximal consumption rate, spare capacity and coupling efficiency were calculated from the recordings of the OCR following addition of different mitochondrial inhibitors (Figure 4c). Oligomycin is an inhibitor of ATP synthase and can be used to determine mitochondrial coupling efficiency, the efficiency with which mitochondria convert oxygen into ATP. CCCP, an uncoupler of mitochondrial oxidative phosphorylation raises OCR to its maximal rate, which allows the calculation of the respiratory reserve. Finally, rotenone and antimycin A, respectively, inhibits the complex I and complex III, which allows the determination of the non-mitochondrial oxygen consumption. Interestingly, a reduced respiratory reserve was observed in cells overexpressing IDH1^R132^ (Figure 4e) or IDH2^R172^ (Supplementary Figure S3B). To determine which complex was involved in the decreased respiratory reserve, OCR was recorded after sequential addition of rotenone and antimycin A. Complex I activity (left panel) contributed for 90% of total OCR, whereas complex II (right panel) contributed for only 10% (Supplementary Figure S4). Mutant IDH did not change the respective contribution of these complexes to mitochondrial respiration. Taken into account that the majority of the electrons entering the electron transport chain (ETC) are doing so at the level of complex I through oxidation of NADH, the decreased mitochondrial respiratory reserve triggered by mutant IDH reflects probably a reduction of the NADH mitochondrial pool. In order to confirm this hypothesis, NADH level was measured in our cells (Figure 4f). Indeed, NADH level was decreased in IDH1^R132^ cells compared with IDH1 cells.

**Figure 5:**
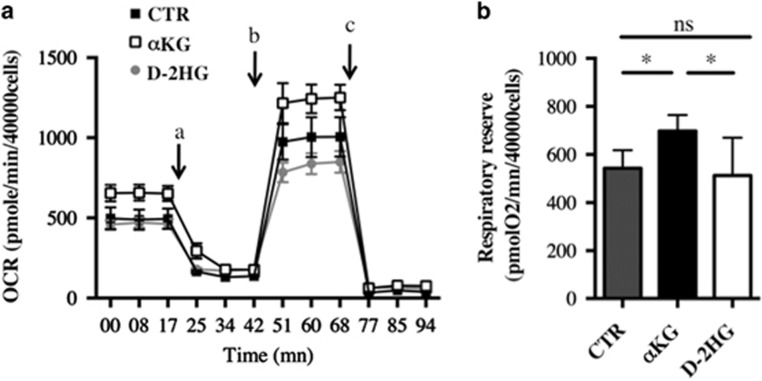
OCR (**a**) and mitochondrial respiratory reserve (**b**) were measured respectively as in Figure 4 in cells treated or not with 5 mM *α*KG or D-2HG for 5 days. Results are expressed as the mean±S.E.M. of three experiments performed in triplicate. *P<0.05, **P<0.01 and ***P<0.001

No difference was observed in the coupling efficiency between ETC activity and ATP synthesis or in glycolysis as reflected by the extracellular acidification rate (ECAR; Supplementary Figure S5). We then assessed the mitochondrial OCR of cells treated with *α*KG or D-2HG for 6 days. The addition of *α*KG significantly increased both the basal and the respiratory reserve, which is not surprising as this metabolite directly fuels the TCA cycle (Figures 5a and b). However, D-2HG affected neither the basal OCR nor the respiratory reserve. Glycolysis was not affected by D-2HG (data not shown).

### Oxamate prevents reduction of both the respiratory reserve and ETO-induced apoptosis triggered by IDH1^R132^

**Figure 6:**
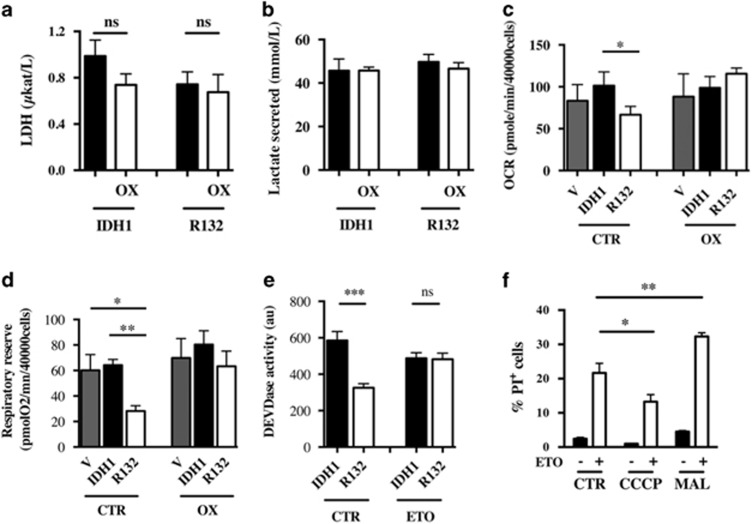
Lactate deshydrogenase activity (**a**) and lactate production (**b**) of U251 cells expressing IDH1 isoforms. Cells were treated or not with 3 mM oxamate (OX) for 48 h. (**c**) Basal oxygen consumption was determined by measuring OCR as in Figure 4c in cells expressing IDH1 isoforms treated or not with 3 mM oxamate for 7 days. (**d**) Mitochondrial respiratory reserve was determined as in Figure 4e in cells treated or not with 3 mM oxamate for 7 days. (**e**) Caspase 3 activation was determined using a DEVDase activity assay 24 h after ETO-induced apoptosis in U251 cells treated or not for 7 days with 3 mM oxamate. (**f**) The number of dead cells was measured 24 h after a concomitant treatment with CCCP (1 *μ*M) or malate (3 mM) and ETO (50 *μ*g/ml). The number of dead cells was determined by FACS after propidium iodide (1 *μ*g/ml) staining. Results are expressed as the mean±S.E.M. of three experiments performed in triplicate

Oxamate is a known inhibitor of lactate dehydrogenase. In order to determine whether forcing the cell into oxidative phosphorylation could affect the sensitivity to apoptosis after ETO treatment, cells were exposed to oxamate (3 mM for 3 days) before metabolic analysis or ETO treatment. Surprisingly, lactate dehydrogenase (LDH) activity was not inhibited by oxamate as shown in Figures 6a and b. Furthermore, although oxamate did not affect basal OCR or the respiratory reserve of vector and IDH1-overexpressing cells, the reduction in both basal OCR and the respiratory reserve observed in overexpressing IDH1^R132^ cells was restored by oxamate (Figures 6c and d). ETO-induced apoptosis was then evaluated in the presence of oxamate. Interestingly, caspase-3 activation was not modified with oxamate after ETO treatment in IDH1-overexpressing cells (Figure 6e). However, the sensitivity of IDH1^R132^ cells to ETO in the presence of oxamate was comparable to that of IDH1 cells (Figure 6e). Altogether, these results strongly suggest that resistance to ETO-induced apoptosis is associated with a depleted mitochondrial NADH pool triggered by mutant IDH expression. In order to determine whether the mitochondrial NADH level can affect cell sensitivity to ETO, U251 cells were treated concomitantly with either CCCP (1μM) or malate (5 mM) and ETO and cell death was analyzed 24 h later. Indeed, although addition of CCCP was associated with reduced cell death, addition of malate led to increased cell death (Figure 6f).

## Discussion

One of the main consequences triggered by tumor-associated IDH mutations is D-2HG accumulation as mutant IDH leads to the synthesis of D-2HG instead of *α*KG.^9^ In this paper, we show that mutant IDH overexpression increases cell proliferation of glioma cells and reduces ETO-induced apoptosis. Although the metabolite D-2HG is involved in the proliferation phenotype, its potential role is excluded for the latter phenotype on cell death. This is the first study showing that D-2HG does not fully recapitulate the IDH mutation phenotype.

The oncogenic potential of D-2HG has been extensively studied since the discovery of IDH mutations. In fact, D-2HG and *α*KG are structurally similar, suggesting that D-2HG may act as a competitive inhibitor of a number of *α*KG-dioxygenases. This hypothesis has been confirmed *in vitro* as the addition of D-2HG to cells causes the inhibition of multiple *α*KG-dioxygenases.^14^ Epigenetic profiling of AML and glioma patient cohorts show that IDH mutations trigger a global DNA hypermethylation.^16, 17^ Several studies have shown that D-2HG is sufficient to disrupt TET2 function and impair histone demethylation.^11, 13, 18^ In addition, this metabolite impacts a number of cellular processes such as increasing cell proliferation and blocking cell differentiation.^18, 19^ In agreement with these reports, we show an increase in cell proliferation at both low and high densities with either mutant IDH or D-2HG treatment. However, other groups^20^ have shown reduced growth with overexpression mutant IDH, generally associated with increased oxidative stress. These data suggest that metabolic abnormalities associated with mutant IDH are cell type specific.

IDH mutations cause many other metabolic abnormalities besides D-2HG accumulation.^21^ Cells expressing mutant IDH exhibit decreased NADPH because of the lack of conversion of isocitrate to *α*KG and also because of the consumption of NADPH in the conversion of *α*KG to D-2HG. NADPH serves as an electron carrier for the maintenance of redox homeostasis and reductive biosynthesis with separate cytosolic and mitochondrial pools. Thus, mutant IDH may lead to increased intracellular ROS oxidation. However, in our cell model, we show that mutant IDH does not affect the total cellular ROS nor affect superoxide dismutase 2 and catalase expression (data not shown). Studies by Leonardi *et al.*^11^ have indicated that mutant IDH may compromise the ability of this enzyme to catalyze the Gln-dependent reductive carboxylation reaction. This pathway can be stimulated by a perturbation in the redox ratio. However, our mutant IDH cells show no selective sensitivity to Gln metabolism inhibitors such as EGCG and BPTES (Supplementary Figure S6). In our model, oxidative stress is not increased with the overexpression of mutants IDH, which could explain why there was no increase in Gln dependency in our glioma cell lines.

Several studies have been published showing opposite effects of mutant IDH on cell death. We show that mutant IDH increases the resistance of gliomas to specific cell death stimuli. Our results are in agreement with the data published by the group of Park, showing an increased sensitivity to radiation in different cell types when IDH1 or IDH2 is silenced.^22, 23, 24, 25^ However, other studies show increased cell sensitivity to cell death when mutant IDH is overexpressed, in particular in ROS-mediated cell death such as radiation-^26^ or BCNU-induced cell death.^27^ However, these cells are addicted to glutaminolysis contrary to the glioma cell lines used in our study. This difference in metabolism could explain the discrepancy observed in cell death sensitivity between the different cell lines. Another explanation for these opposing results could be the specific genetic background of the different cell types, in particular the expressed forms of p53 and EGFR, the methylation status of MGMT and the presence or not of PTEN. This raises the possibility that, in general, the phenotype associated with mutant IDH may be restricted to some cell lines. Mutant IDH may also increase the resistance of gliomas to specific cell death stimuli. Resistance to apoptosis by IDH1^R132^ provides a rationale for the high frequency of IDH1^R132^ in secondary GBM.

**Figure 7:**
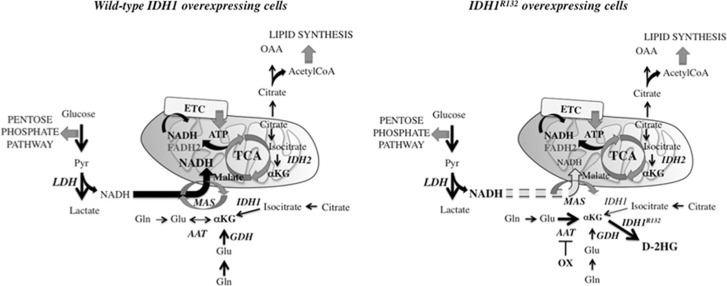
Metabolic alterations driven by IDH mutation in U251 cells. In cancer cells, glycolysis upregulation generates the production of reducing equivalents NADH, which is then shuttles from the cytosol to mitochondria with the MAS. Glutamine is converted to glutamate, which is further converted to *α*KG by GDH. IDH also produces *α*KG from isocitrate. In cells expressing IDH1^R132^, IDH1^R132^ converts *α*KG into D-2HG. To limit cellular *α*KG depletion, IDH1^R132^-overexpressing cells diverts AAT from MAS function to produce *α*KG. As a result, glycolytic NADH is no longer fully imported into mitochondria. AAT, aspartate aminotransferase; ETC, electron transport chain; GDH, glutamate dehydrogenase; Gln, Glutamine; Glu, Glutamate; IDH, isocitrate dehydrogenase; LDH, lactate dehydrogenase; MAS, malate–aspartate shuttle; Pyr, pyruvate; TCA, tricarboxylic acid cycle

Interestingly, our data show that both mutant IDH1 and mutant IDH2 that, respectively, are expressed in cytosol and mitochondria, lead to decreased mitochondrial respiratory reserve. Numerous studies have shown that IDH mutants use *α*KG as a substrate to produce D-2HG instead of producing it. This drain of *α*KG must be balanced to some extent by the cells. There are two ways of converting glutamate (Glu) into *α*KG, either by deamination through glutamate dehydrogenase (GDH) or by transamination through aspartate aminotransferase (AAT).^28^ Usually, AAT functions in tandem with the malate dehydrogenase (MDH) in the malate–aspartate shuttle (MAS), which transfers reducing equivalent NADH from the cytosol to mitochondria. Glycolysis, which is highly upregulated in cancer cells, is a key source of the reduced form of cytosolic NADH, mainly at the level of LDH.^29^ As a result, the activity of MAS is increased to shuttle the glycolytic NADH into mitochondria.^30^ However, it has also been shown that the presence or the lack of mitochondrial substrates could greatly influence the ability of AAT to effectively compete with GDH for Glu.^31^ We speculate that to limit cytosolic and mitochondrial *α*KG depletion induced by the presence of mutant IDH, cells are using AAT, independently of MDH, to produce *α*KG rather than to shuttle NADH in mitochondria (Figure 7). As a result, the dissociation of AAT activity from MDH results in the accumulation of the reductive power of malate trapped in the cytosol and the reduction of mitochondrial NADH pool built from the MAS activity. Altogether, these effects lead ultimately to a reduced mitochondrial respiratory reserve. Further experiments such as direct inhibition of AAT need to be performed in order to confirm our hypothesis. However, it is in agreement with metabolomic studies showing that mutant IDH is associated with decreased fumarate and malate levels, as well as mitochondrial dysfunction.^32^ Furthermore, it is reinforced with the recovery of the mitochondrial respiratory reserve with oxamate. Indeed, besides being an inhibitor of LDH, oxamate also inhibits AAT.^33, 34^ In our study, oxamate did not decrease lactate secretion as predicted by its LDH inhibitory action but instead prevented the mitochondrial respiratory reserve loss only in mutant IDH-overexpressing cells.

This metabolic alteration has an important role in the resistance to ETO-induced apoptosis in IDH^R132^-overexpressing cells. ETO is a DNA-topoisomerase II inhibitor widely used in the treatment of diverse tumors.^35^ Like many other agents, ETO can be used in monotherapy or in combination with other treatments. One of the major pathways of ETO metabolism in cells involves cytochrome P450 reductase, which uses NADPH as a cofactor for its enzymatic activity. Furthermore, it has been shown that ETO-induced apoptosis requires fully functional mitochondria.^36^ Our data and others show that mutant IDH decreases the cellular NADPH pool combined with the altered mitochondrial metabolism^37^ may explain the specific resistance to ETO of IDH1^R132^-overexpressing cells.

Collectively, our data support the hypothesis that mutant IDH affects tumor progression and therapy resistance, which may, at least in part, explain the high frequency of IDH mutation in secondary GBM. On the basis of our and other observations, D-2HG mediates pro-tumorigenic effects, whereas altered metabolism and in particular the imbalance of NAD+ and NADP+ coenzymes are associated with cell resistance to specific cell death stimuli.

In conclusion, IDH mutation deregulates mitochondrial metabolism and as a consequence alters cell sensitivity to specific stimuli. Clinically, a better understanding of IDH mutations will enable IDH-directed therapies to be developed in the future.

## Materials and Methods

### Cell culture and stable transfection

Glioblastoma U251 and LN18 cell lines were cultured at 37 °C in a humidified atmosphere with 5% CO_2_ in Dulbecco's modified Eagle's medium (DMEM) containing 5 g/l glucose and supplemented with 100 U/ml penicillin, 100 *μ*g/ml streptomycin, 2 mM Gln and, respectively, 10% and 5% fetal calf serum (FCS). T98 cells were cultured in DMEM 1 g/l glucose, supplemented with penicillin, streptomycin, Gln and 10% FCS. Cells were seeded in 12-well plates and transfected with Lipofectamine 2000 transfection reagent as recommended by the manufacturer (Life Technologies, Carlsbad, CA, USA). The expression vectors for wild-type and mutated IDH1 and IDH2 were described previously.^38^ U251, LN18 and T98 stable cells lines overexpressing IDH were selected using geneticin (Life Technologies). Hydroxyglutarate was purchased from Peptech (Bedford, MA, USA) and *α*KG, dimethyl-*α*KG and oxamate from Sigma (St Louis, MO, USA). For cell death experiments, 0.5 × 10^6^ cells were plated and treated the next day with 50 *μ*g/ml ETO (Mylan, St Priest, France), 50 ng/ml TRAIL (PreproTech, Neuilly sur-Seine, France), 60ng/ml FASL (PreproTech) and 15 *μ*g/ml cisplatin (Mylan). *γ*-Irradiation was carried out in a Faxitron CP160 irradiator (Faxitron X-ray Corporation, Tucson, AZ, USA) at a dose rate of 5 Gy. The number of dead cells was evaluated by FACS after incubation of 5 min with propidium iodide (Sigma).

### Cell counts, viability and clonogenicity assay

Cell counts and viability were performed using the Countess optics and image automated cell counter (Life Technologies). Cells were mixed with trypan blue (50/50) and loaded into a Countess chamber slide. The image analysis software was used to automatically analyze the acquired cell images from the sample to give cell count and viability. Data were plotted either as the number of viable cells for cell proliferation or as the percent of dead cells for viability assessment. For cell proliferation, cells were seeded at 100.000 cells and counted every 3 to 4 days. For the clonogenicity assay, 500 cells were seeded on a 6-well plate. After 1 week, cells were fixed and stained with a solution of violet crystal 0.05% in ethanol 50%. The number of colonies is counted.

### ROS and mitochondrial membrane potential

ROS production was measured using a DCFDA probe (Life Technologies). Cells were seeded at 2. × 10^4^ cells in a 96-well plate and incubated with the DCFDA probe. The fluorescence is measured at 538 nm every 3 min for 75 min. Mitochondrial membrane potential was evaluated by FACS analysis using JC-1 probe (Life Technologies) staining accordingly to the manufacturer's instruction.

### Biochemical analysis

Production of NADH and NADPH was measured using, respectively, the NAD/NADH kit (Abcam, Cambridge, UK) and the NADP/NADPH kit (Abcam) according to the manufacturer's instruction. Lactate and LDH activity were measured using the Roche diagnostic kit on a Cobas 8000 (Roche Diagnostics, Mannheim, DE, USA) as described previously.^39^

### Protein lysates, immunoblotting and caspase activation

Cells were lysed at the indicated time points in RIPA lysis buffer (25 mM Tris-HCl, pH 7.6 containing 150 mM NaCl, 1% NP40, 1% Na-deoxycholate and 0.1% SDS) supplemented with protease inhibitors. Protein concentration was determined using BCA protein assay (Sigma). Samples were adjusted accordingly, analyzed by SDS-PAGE and subsequent immunoblotting with antibodies that recognize IDH1 (Abcam), IDH2 (Abcam), IDH1-R132H (Dianova, Hamburg, Germany), actin (Millipore, Billerica, MA, USA), Bax (BD Pharmingen, San Jose, CA, USA), Bcl2 (BD Pharmingen), XIAP (R&D system, Abingdon, UK), tBid (R&D System), porin (Calbiochem, Nottingham, UK), survivin (Cell Signaling Technology, Danvers, MA, USA). HRP-conjugated secondary antibodies were from BioRad (Marnes-la-Coquette, France). The ImageJ 1.42q software (NIH) was used to digitally quantify the signal intensities of western blot bands. Caspase 3 activity was quantified using the fluorogenic substrate Ac-DEVD-AMC, as described in.^15^

### OCR and extracellular consumption rate (ECAR)

Cells were plated at 40 000 cells per well in 24-well XF (extracellular flux) cell culture microplate (Seahorse Bioscience, Copenhagen, Denmark). OCR was measured the next day using a XF24 Analyzer (Seahorse Bioscience). Cells were equilibrated for 1 h at 37 °C in bicarbonate-free DMEM (Sigma) supplemented with 25 mM glucose, 1 mM pyruvate and 2 mM Gln, pH 7.3 before any measurement. To determine mitochondrial parameters, OCR was measured at baseline and after addition of oligomycin (0.4 *μ*M), FCCP (2 *μ*M), rotenone (0.6 *μ*M) and antimycin A (0.6 *μ*M). All measurements were done at least in three wells per condition, twice for each experiment. Respiratory reserve was calculated as maximal OCR, after oligomycin and CCCP injections, minus basal OCR. Coupling efficiency corresponded to OCR inhibition by oligomycin. Relative contribution of complex I and II to OCR corresponded, respectively, to OCR inhibition by rotenone and antimycin A.

### Statistical analysis

Experiments were done at least three times and data were analyzed using GraphPad Prism 5.00 (GraphPad Software, San Diego, CA, USA). Differences with *P*-values<0.05 were considered statistically significant.

## Figures and Tables

**Table 1:** Cell death was monitored in U251 after irradiation (5 Gy), ETO (50 *μ*g/ml), TRAIL (50 ng/ml), FasL (60 ng/ml) or cisplatin (15 *μ*g/ml) treatment after 5, 24, 48 and 72 h by FACS analysis using propidium iodide incorporation and caspase-3 activation

	**CTR**	**6 h**	**24 h**	**48 h**	**72 h**
*Etoposide*					
% Dead cells	2.6±0.8 (*n*=3)	NA	28±2 (*n*=4)	28±3 (*n*=4)	NA
DEVDase activity	3.3±0.3 (*n*=3)	37±11 (*n*=4)	204±18 (*n*=4)	128±11 (*n*=4)	NA
					
*Irradiation*					
% Dead cells	2.6±0.8 (*n*=3)	NA	6±2 (*n*=3)	8±1 (*n*=3)	15±1 (*n*=5)
DEVDase activity	3.3±0.3 (*n*=3)	NA	65±25 (*n*=3)	NA	143±13 (*n*=5)
					
*TRAIL*					
% Dead cells	2.6±0.8 (*n*=3)	22±3 (*n*=4)	6.4±1 (*n*=3)	NA	NA
DEVDase activity	3.3±0.3 (*n*=3)	107±3 (*n*=4)	7±0.3 (*n*=3)	NA	NA
					
*FASL*					
% Dead cells	2.6±0.8 (*n*=3)	10±1 (*n*=3)	20±3 (*n*=3)	NA	NA
DEVDase activity	3.3±0.3 (*n*=3)	38±8 (*n*=3)	115±3 (*n*=3)	51±13 (*n*=3)	NA
					
*Cisplatin*					
% Dead cells	2.6±0.8 (*n*=3)	NA	16±2 (*n*=3)	NA	NA
DEVDase activity	3.3±0.3 (*n*=3)	NA	139±18 (*n*=3)	NA	NA

Abbreviation: NA, not applicable

Apoptosis was measured through caspase-3 activation

## Abbreviations

*α*KG: alpha ketoglutarate
AAT: aspartate aminotransferase
D-2HG: D-2-hydroxyglutarate
ECAR: extracellular acidification rate
ETC: electron transport chain
ETO: etoposide
GBM: glioblastoma multiforme
Gln: glutamine
Glu: glutamate
IDH: isocitrate dehydrogenase
OCR: oxygen consumption rate
