# Supplementary figures and images for: D-2-Hydroxyglutarate does not mimic all the IDH mutation effects, in particular the reduced etoposide-triggered apoptosis mediated by an alteration in mitochondrial NADH
Source: Cell Death Dis. 2015 Mar 26;6(3):e1704–. doi: 10.1038/cddis.2015.13 (PMC4385911; doi:10.1038/cddis.2015.13)

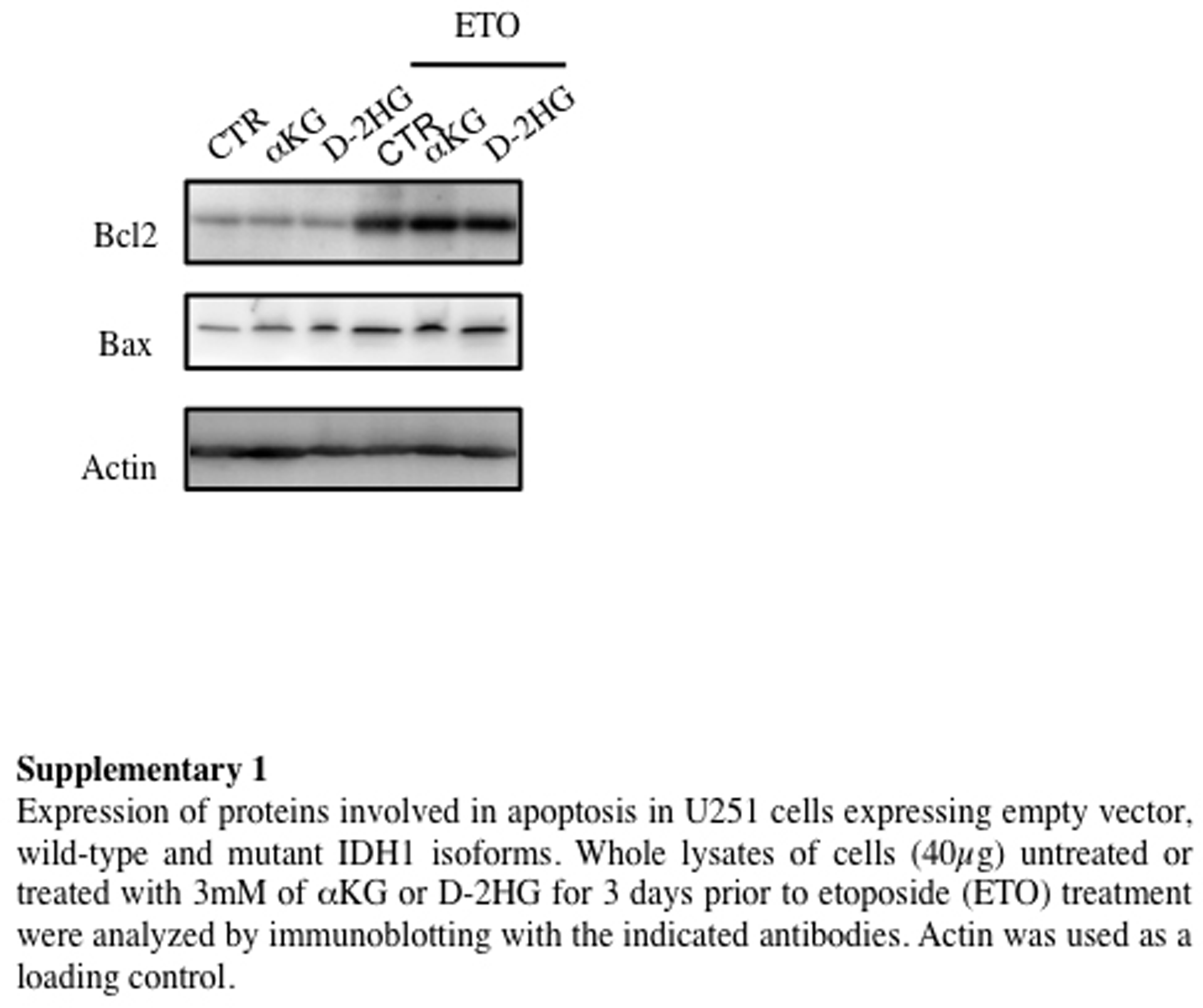

Supplement: Supplementary Figure S1 [file cddis201513x1.tif]

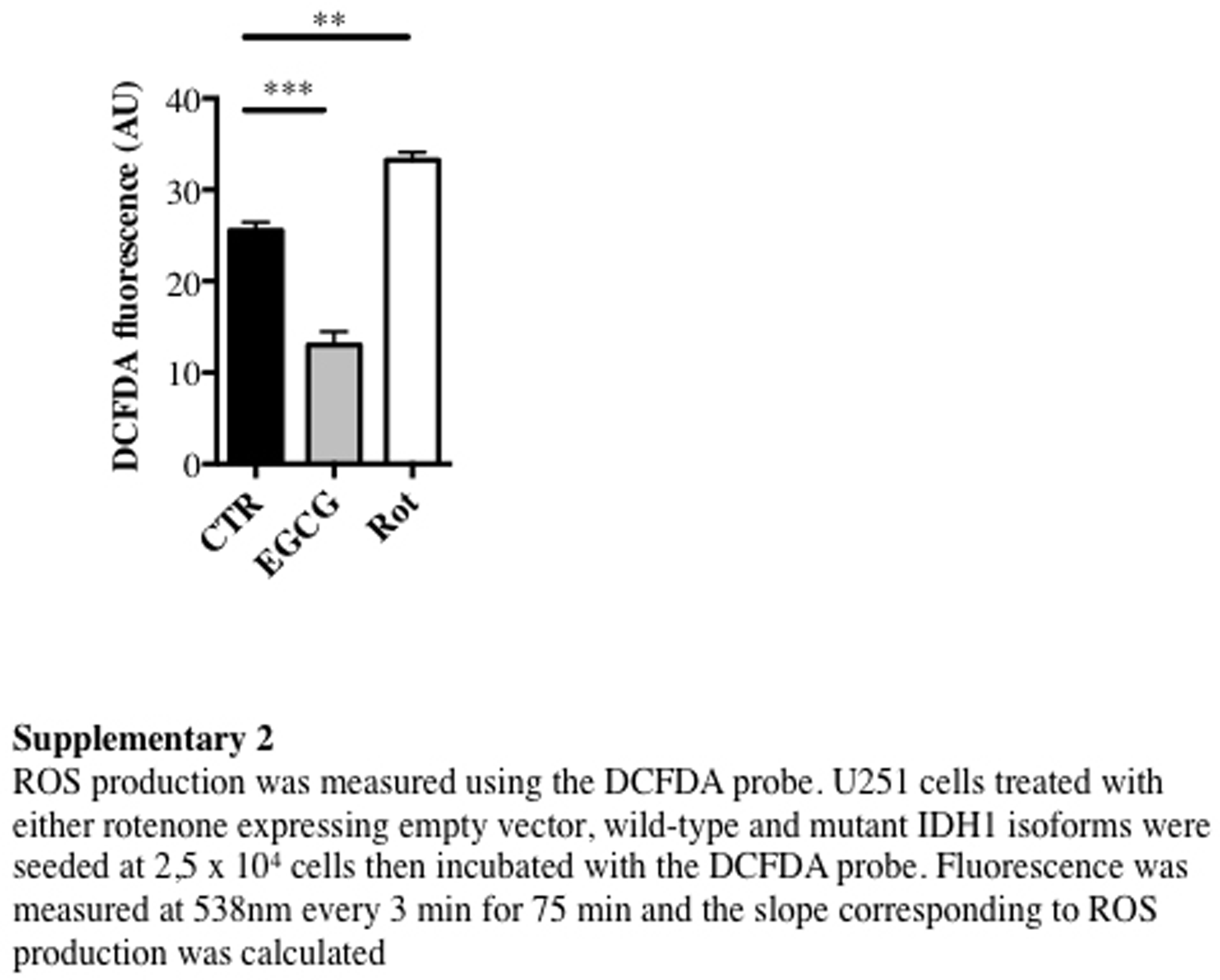

Supplement: Supplementary Figure S2 [file cddis201513x2.tif]

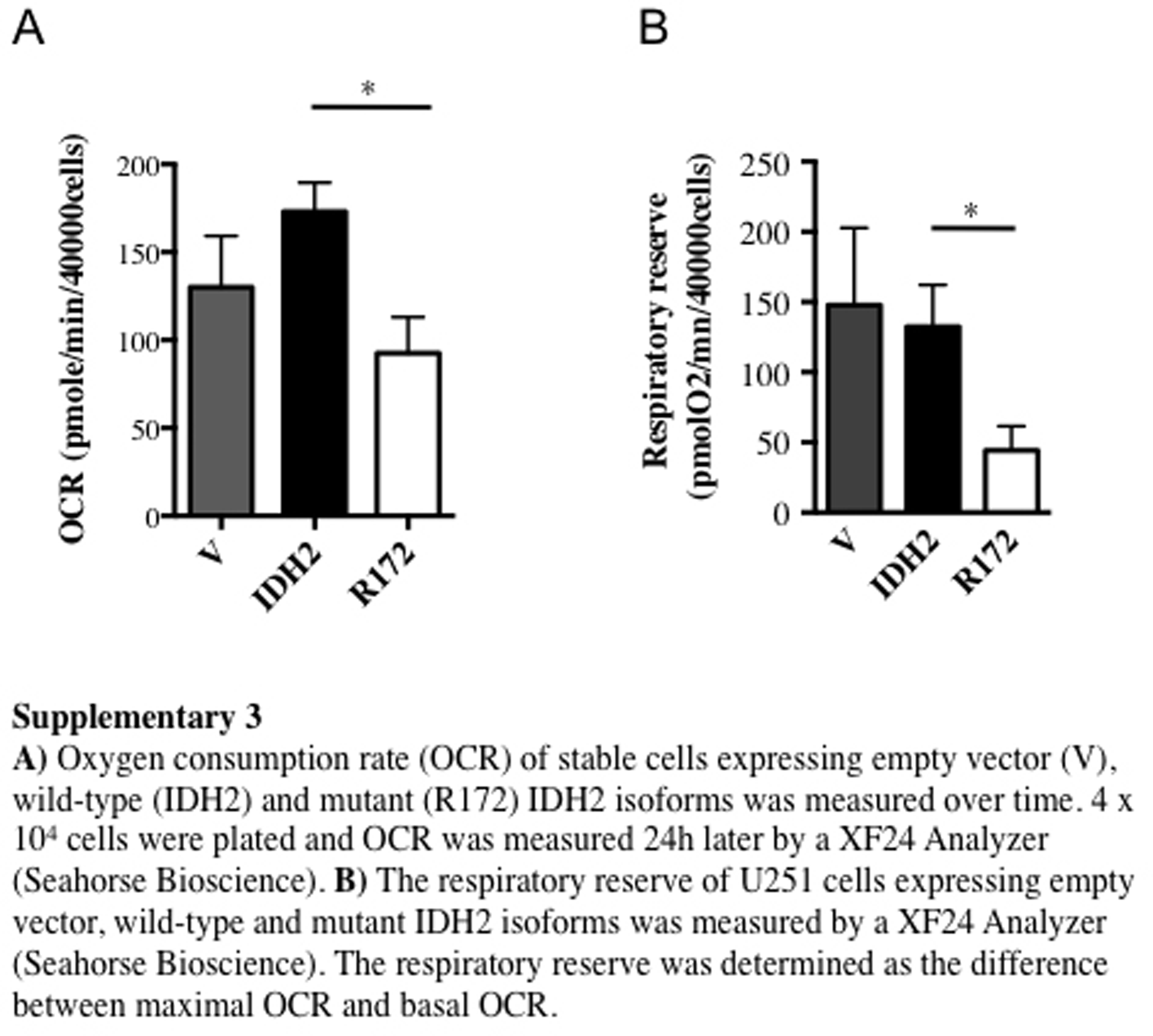

Supplement: Supplementary Figure S3 [file cddis201513x3.tif]

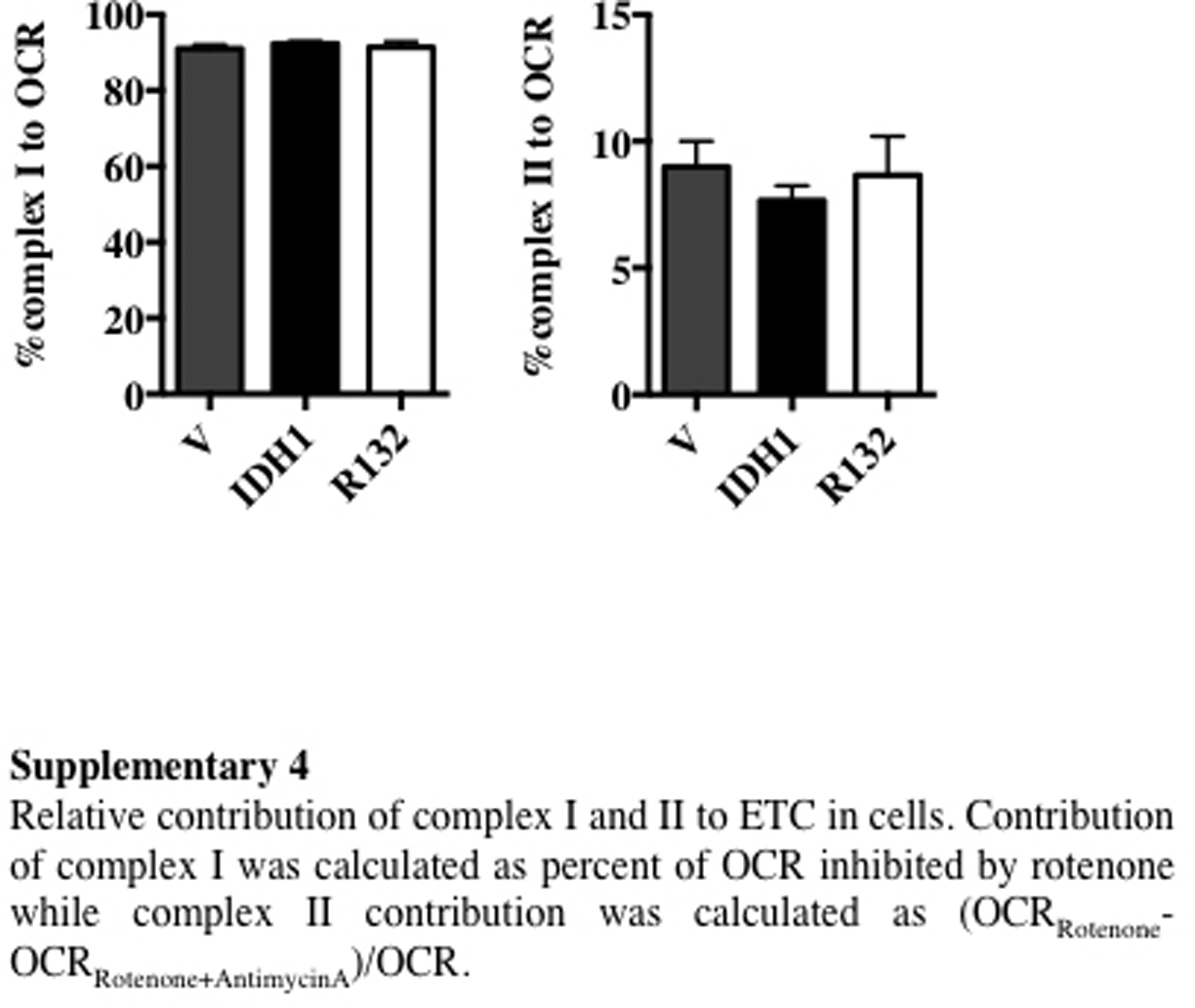

Supplement: Supplementary Figure S4 [file cddis201513x4.tif]

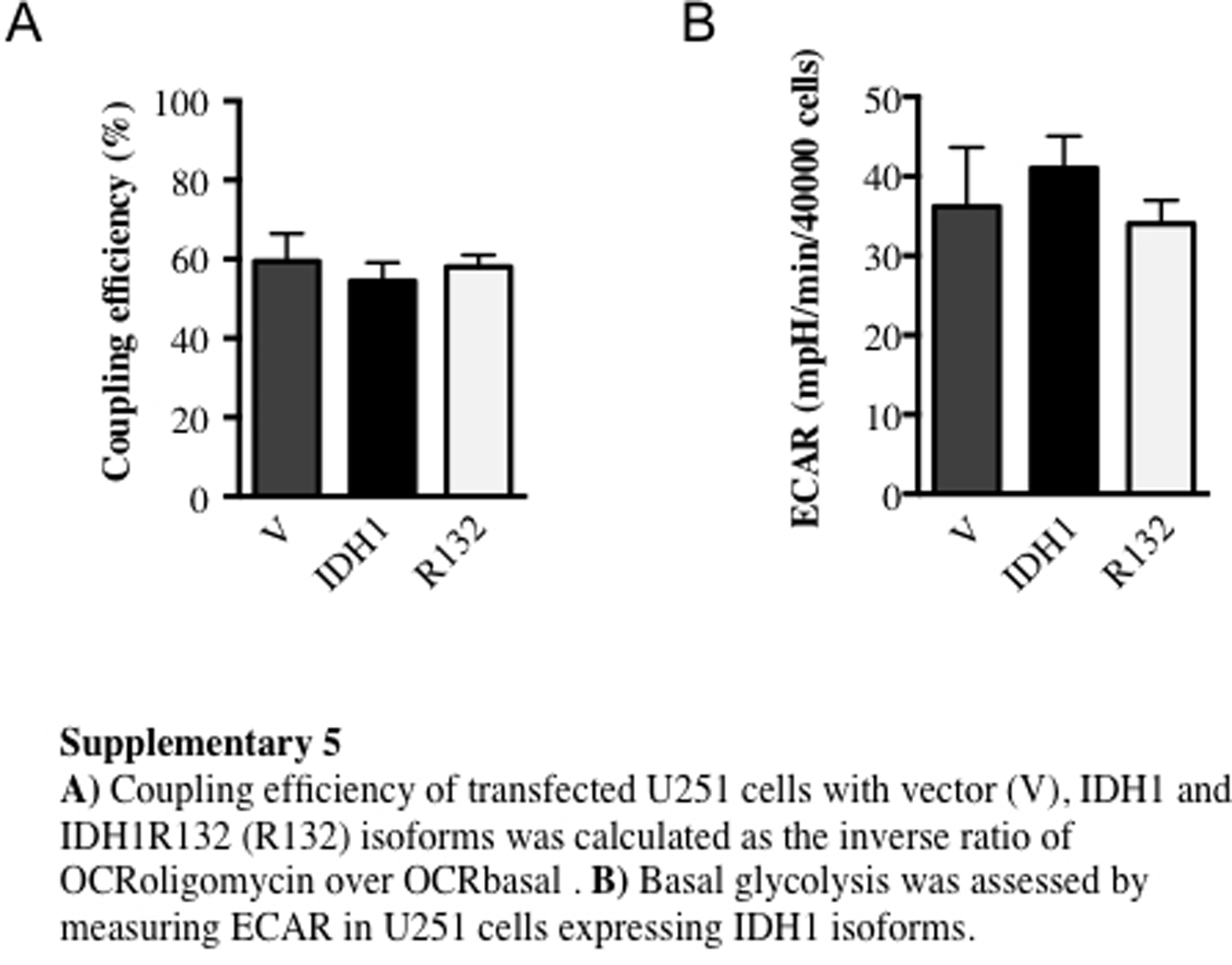

Supplement: Supplementary Figure S5 [file cddis201513x5.tif]

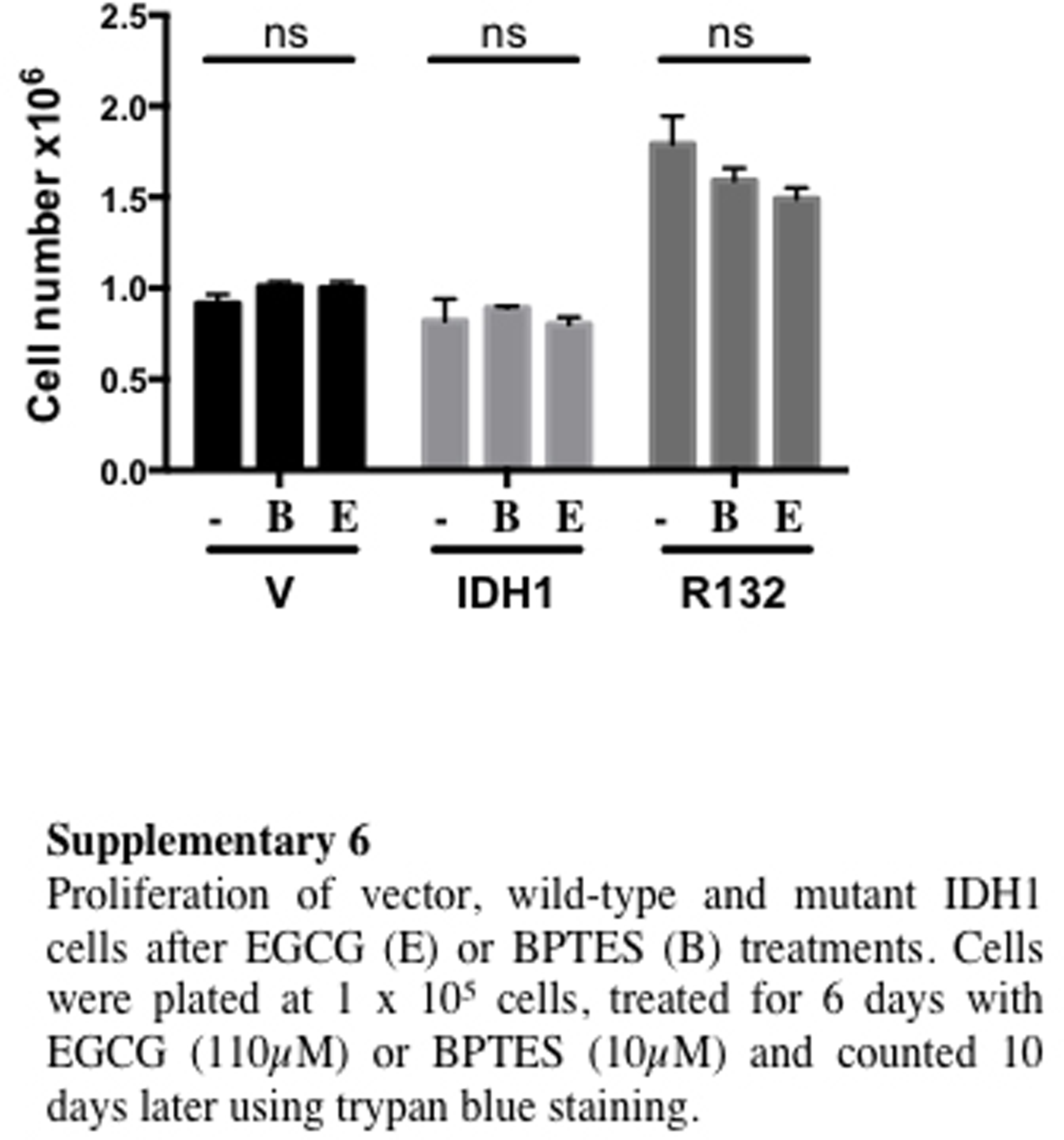

Supplement: Supplementary Figure S6 [file cddis201513x6.tif]
